# Q-DOAS: A Proximity Quenching Assay for Real-Time Detection of Early Protein Aggregation Events

**DOI:** 10.64898/2026.06.16.732424

**Published:** 2026-10-21

**Authors:** Korbin Michael Kleczko, Dan Gestaut, Sarah Dobbins, Juliana Abramovich, Cole Sitron, Xia Li, Rebecca Chan, Nan Wang, X. William Yang, F. Ulrich Hartl, Judith Frydman

## Abstract

Protein aggregation is central to numerous neurodegenerative diseases. The paucity of facile assays to measure aggregation and detect amyloid seeds hinders therapeutics development and clinical assessments. The standard ThT assay reports on amyloid formation but is blind to early oligomers and is prone to interference. We describe Q-DOAS, a simple and reproducible assay that quantifies aggregation in real time via proximity-quenching of a site-specifically conjugated dye (BODIPY-TMR). Using mutant Huntingtin-exon 1 (mHTT-Ex1) and α-Synuclein A53T, we show Q-DOAS detects pre-amyloid oligomers, yielding quantitative kinetic data compatible with mechanistic analysis. We further demonstrate its utility to dissect mutational effects, screen for protein and small-molecule aggregation inhibitors, and quantify amyloid seeding activity in cellular and mouse models of Huntington’s disease and in cerebrospinal fluid from Parkinson’s disease patients without amplification. Q-DOAS provides a sensitive, robust, and scalable tool for studying the earliest events in amyloid pathologies and for advancing therapeutic development.

